# Genetic Analysis of Infectious Bronchitis Virus S1 Gene Reveals Novel Amino Acid Changes in the GI-16 Lineage in Peru

**DOI:** 10.3390/microorganisms11030691

**Published:** 2023-03-08

**Authors:** Eliana Icochea, Rosa González, Gina Castro-Sanguinetti, Lenin Maturrano, Luis Alzamora, Luiz Sesti, Jorge Chacón, Juan More-Bayona

**Affiliations:** 1 Laboratory of Avian Pathology, Faculty of Veterinary Medicine, Universidad Nacional Mayor de San Marcos, Av. Circunvalación 2800, Lima 15081, Peru; 2 Laboratory of Genetics, Faculty of Veterinary Medicine, Universidad Nacional Mayor de San Marcos, Av. Circunvalación 2800, Lima 15081, Peru; 3 CEVA Animal Health, 3461 Av. República de Panamá Dpto. 1102, San Isidro, Lima 15047, Peru; 4 Laboratory of Virology, Faculty of Veterinary Medicine, Universidad Nacional Mayor de San Marcos, Av. Circunvalación 2800, Lima 15081, Peru

**Keywords:** avian coronavirus, infectious bronchitis virus, infectious bronchitis, GI-16 lineage, Q1, GI-1 lineage

## Abstract

Infectious bronchitis is a highly contagious viral disease that represents an economic threat for poultry despite the wide use of vaccination. To characterize the virus circulating in Peru, we analyzed 200 samples, including nasopharyngeal swabs and multiple tissues collected from animals suspected of being infected with infectious bronchitis virus (IBV) between January and August in 2015. All animals had at least one positive sample for IBV by RT-PCR. Out of these positive samples, eighteen (18) were selected for viral isolation and a partial S1 sequencing. Phylogenetic analysis showed that sixteen isolates clustered with members of GI-16 lineage, also known as Q1, with nucleotide homology ranging from 93% to 98%. The two remaining isolates grouped with members of the GI-1 lineage. Our study reveals circulation of GI-16 lineage during this period in poultry systems in Peru, along with GI-1 lineage (vaccine-derived). Moreover, those IBV GI-16 isolates showed unique nucleotide and amino acid changes compared to their closest relatives. Altogether, these findings reveal the circulation of GI-16 lineage while describing changes at key regions of the S protein that might be of relevance for vaccine evasion. These results highlight the importance of genetic surveillance for improving vaccination strategies against infectious bronchitis.

## 1. Introduction

Infectious bronchitis (IB) is a highly contagious viral disease that mainly affect the respiratory system leading to a major impact on poultry despite the implementation of vaccination programs [1,2,3]. IB is caused by an avian coronavirus (*Coronaviridae* family, *Orthocoronavirinae* subfamily, *Gammacoronavirus* Genus and *Igacovirus* subgenus), commonly known as infectious bronchitis virus (IBV). Its genome, a single-stranded RNA, positive sense of 27.6-kb, encodes four structural proteins (S, M, N and E) and a large polyprotein 1a and 1ab, including multiple accessory proteins [4,5]. Due to its viral replication properties, multiple serotypes and lineages have emerged attributed to its high genetic diversity [6,7]. Regardless of this genetic variation, strains of GI-1 lineages, such as Massachusetts strain, continues to be one of the most used in the vaccine development due to its worldwide distribution. On the other hand, Q1 genotype members (GI-16) that originally emerged in China in the middle of the 20th century, distributed into Asia, Middle East, and Europe, are recently becoming a major concern in some South American countries [8].

In South America, GI-16 lineage has been identified in Argentina, Chile, Uruguay, and Colombia [9,10]. More recently, genetic studies have suggested recombination events of circulating IBV in Peru and Uruguay, which might reveal the underlying events of viral evolution occurring in South American countries [11,12]. Furthermore, IBV GI-1 lineage has been detected in countries, such as Brazil [13,14,15,16], Argentina [9], Chile [17,18], and Colombia [19]. Although its first detection in South America dated back to 1950s [20], it is possible that circulating GI-1 viruses arose from vaccine strains [9]. This is due to the most used vaccine in South America are those to the GI-1 lineage. In addition, some countries, such as Brazil, have detected their own genotypes [21]. Thus, further studies in IBVs circulating in these countries are needed to better understand the evolution and its contribution to improve vaccination strategies. Hence, we conducted a study for detection, isolation, and IBV genotyping by a sequencing of the partial S1 region for the phylogenetic reconstruction of IBV circulating in Peru during 2015.

## 2. Materials and Methods

### 2.1. Samples

Two hundred (200) broiler chickens with respiratory clinical signs, and suggestive of infectious bronchitis (IB), were submitted to the Laboratory of Certificaciones del Peru (Cerper) and the Laboratory of Avian Pathology from the Faculty of Veterinary Medicine, in the Universidad Nacional Mayor de San Marcos at Lima, Peru. These animals were taken from eight (08) poultry farms, located in the major areas for poultry production in Peru, including Lima, Trujillo, Ica, Arequipa y San Martin. Of note, these samples were obtained from January to August 2015. Samples comprising the trachea, cecal tonsils, and/or kidneys were processed accordingly and analyzed by real-time RT-PCR tests for IBV diagnosis. Sample collection procedure was approved by the Ethics committee of the Faculty of Veterinary Medicine of the Universidad Nacional Mayor de San Marcos (Authorization N° 2021-33).

### 2.2. Molecular Detection

Viral RNA extraction was performed using a QiaAmp viral RNA Mini Kit (Qiagen, Germany) according to manufacturer’s instructions and stored at −80 °C. Following RNA extraction, infectious bronchitis virus was detected using Qiagen One Step RT-PCR Kit (Qiagen, Germany), amplifying the 3′ UTR region of IBV genome [22,23], using a SYBR^®^ green-based assay. The qRT-PCR was performed in a reaction volume of 25 µL containing: 5 µL of RNA (1–2 ug), UTR 41+ and UTR 31- primers (200 nM of each primer, Table 1), master mix (2.5 mM MgCl_2,_ 0.4 mM of each dNTPs), 6.25 µL of ultrapure water and reverse transcriptase (1U). The one-step protocol consists of a reverse transcription at 48 °C per 10 min, followed by an incubation at 95 °C per 3 min; and the amplification was carried out through 40 cycles consisting of denaturation for 5 sec at 95 °C, hybridization for 10 sec at 52 °C and extension for 13 sec at 72 °C. The procedure allows the amplification of a 179 bp segment with a melting temperature (Tm) of 80.1–80.5 °C. Both amplification curve (Ct) and melting curve (Tm) were used as criteria for positive or negative status. Samples showing a low Ct value (Ct < 20) were selected for viral isolation and further partial S1 region amplification and sequencing analysis.

### 2.3. Viral Isolation

Viral isolation was performed from positive samples for IBV detected by PCR with low Ct values (Ct < 20). Supernatant from homogenized tissues and nasopharyngeal swabs were filtered and treated with antibiotics (penicillin and streptomycin) for 2 h. Two hundred microliters were inoculated into the allantoid cavity of 9–11 days-old chicken embryonated eggs. Following inoculation, eggs were maintained at 37 °C for five days and checked daily. Lesions of viral replication in embryos were recorded. Three blinded passages into eggs were performed.

### 2.4. Partial S1 Region Sequencing

S1 characterization has been commonly used for genetic identification of infectious bronchitis virus (IBV), and it has been recently proposed as a tool for phylogenetic classification [24]. The hypervariable region III (HVR-III) of the S1 subunit of S gene was amplified by a conventional RT-PCR reaction according to the protocol previously described [25,26,27], using the primers XCE1 and XCE2 (Table 1). The PCR protocol was performed using 10 µL of a cDNA, 1 µL of XCE1 and XCE2 primers (250 nM of each primer), 5 µL of 10x PCR buffer (1U of Taq polymerase, 2.5 mM MgCl_2_, 0.4 mM of each dNTPs), and 30.5 µL of ultrapure water. The mixture was heated at 94 °C (denaturation) for 5 min and 70 °C for 2 min. PCR cycling consists of 35 cycles of: denaturation at 96 °C for 30 s, annealing for 15 sec at 50 °C, and extension at 60 °C for 4 min. Amplicons of 398 bp were purified using the PureLink™ Quick Gel Extraction and PCR Purification Combo Kit (Invitrogen, USA) according to the manufacturer’s instructions. The sequencing reactions were performed using a Big Dye Terminator V3.1 Cycle Sequencing Kit (Applied Biosystems, Carlsbad, CA USA) with the same primers set for RT-PCR S1 HVR and sequenced in Genetic Analyzer Applied Biosystems 3130 (ABI, Austin, TX, USA). Sequences obtained were used for phylogenetic analysis. Sample types and IDs of viral isolates selected for sequencing is shown in Table 2.

### 2.5. Phylogenetic Analysis

The S1 infectious bronchitis virus sequences were compared with others available on the National Center for Biotechnology Information (NCBI) Infectious Bronchitis Viruses Resource. General information of sequences used in the analysis is shown in Table A1. We performed a multiple sequence alignment using ClustalW from MegaX software [28]. We included in the analysis representative sequences from group 1 of infectious bronchitis virus lineages (*n* = 50) and two representative sequences of genotype II (*n* = 2). The evolutionary analysis was inferred by a maximum likelihood method. A discrete gamma distribution was used to model evolutionary rate differences among sites. Bootstrap analysis was carried out on 1000 data sets.

## 3. Results

### 3.1. IBV Was Detected in Samples Suspected of Infectious Bronchitis

**Figure 1:**
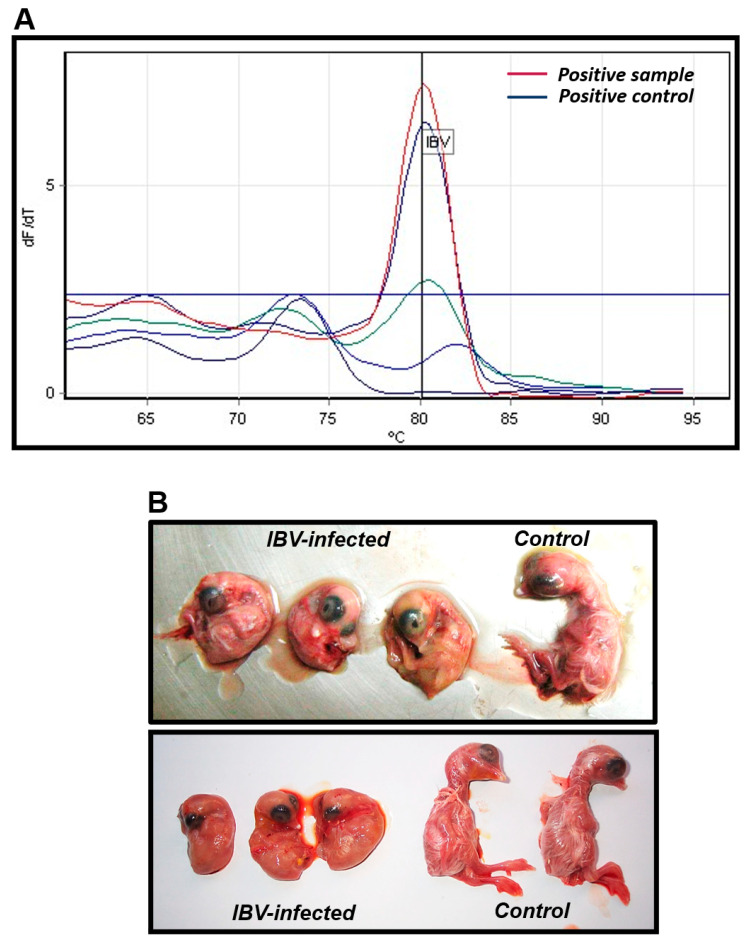
Infectious bronchitis virus was detected and isolated as cause of respiratory disease. Samples submitted for diagnosis were tested by real time RT-PCR for detection of infectious bronchitis virus. (**A**) A representative image of a positive sample is shown. (**B**) Positive samples with a Ct value lower than 20 were selected for isolation into chicken embryonated eggs. Typical lesions were registered. A representative image of embryos showing dwarfing and curling, typical of IBV replication, is presented.

Molecular assays based on genetic amplification are highly sensitive and specific methods for pathogen detection. We took advantage of this to amplify the 3′ UTR segment of IBV by a SYBR^®^ green-based real-time PCR platform. Thus, the infectious bronchitis virus was detected in samples suspected of IBV infection. Despite the heterogeneity in tissue samples obtained, at least one sample per bird was positive for IBV in all samples analyzed. A representative image of a real time PCR-positive sample is shown in Figure 1A. Positive samples had a Tm value within 80.1 and 80.5 °C, while Ct values ranged from 7–35. Samples with a Ct value lower than 20 were selected for viral isolation and further sequencing analysis. Thus, we selected eighteen (18) samples for isolation.

### 3.2. Infectious Bronchitis Virus Was Successfully Isolated in Embryonated Eggs

Selected samples were inoculated into chicken embryonated eggs to amplify viral load. Typical lesions of viral replication were characteristics of IBV replication in embryos, such as dwarfing, curling, hyperemia, among others. Representative images are shown in Figure 1B. Allantoid fluid was harvested for identification by IBV lineages circulating in these areas, based on the characterization of a partial S1 region. Sequences had good quality with both primers in the forward and reverse positions, and the consensus sequences obtained were used to perform the phylogenetic analysis. A file including all 18 sequences is shown (Supplementary S1).

### 3.3. Partial S1 Region Sequencing Reveals IBV GI-16 and -1 Circulation in Peru

**Figure 2:**
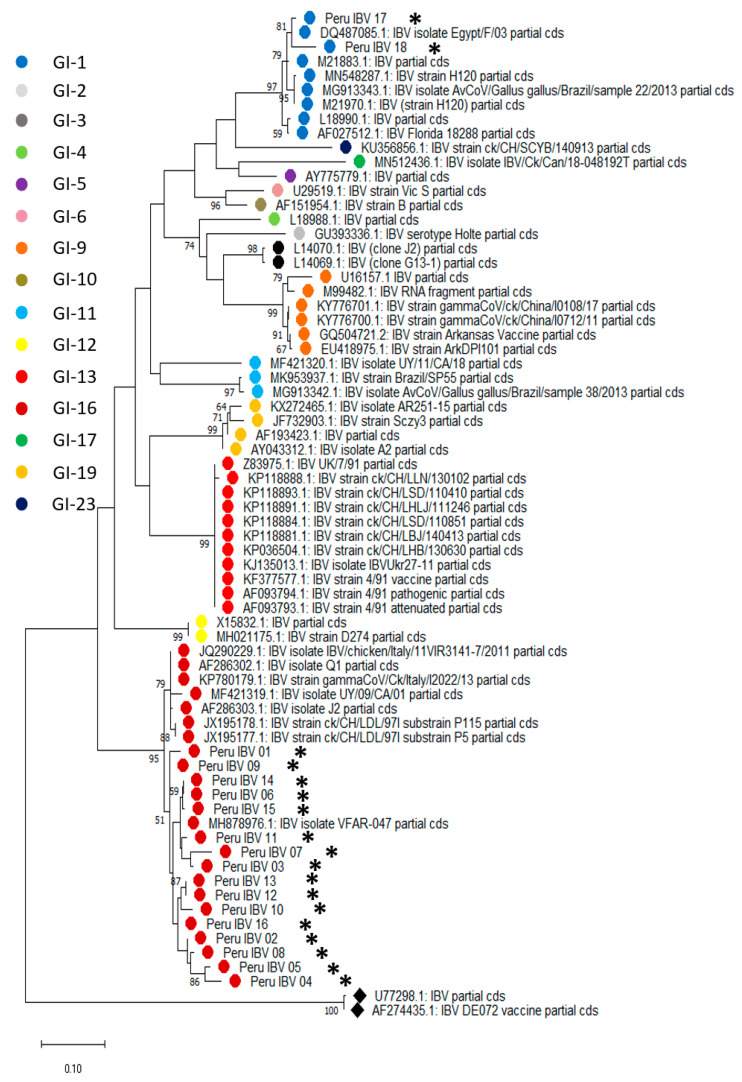
Phylogenetic analysis reveals GI-16 and GI-1 circulation in poultry farms in Peru. Isolated samples were processed for RNA extraction and a PCR that targeted the hypervariable region III (HVR-III) allowed the identification of GI-16 and GI-1 lineages in Peru. The evolutionary history was inferred by using the maximum likelihood method and tamura 3-parameter models. The percentage of trees in which the associated taxa clustered is shown next to the branches. A discrete gamma distribution was used to model evolutionary rate differences among sites. The tree is drawn to scale, with branch lengths measured in the number of substitutions per site. This analysis involved 70 nucleotide sequences. Evolutionary analyses were conducted in MegaX. Asterisks represent the sequences obtained in the current study.

**Figure 3:**
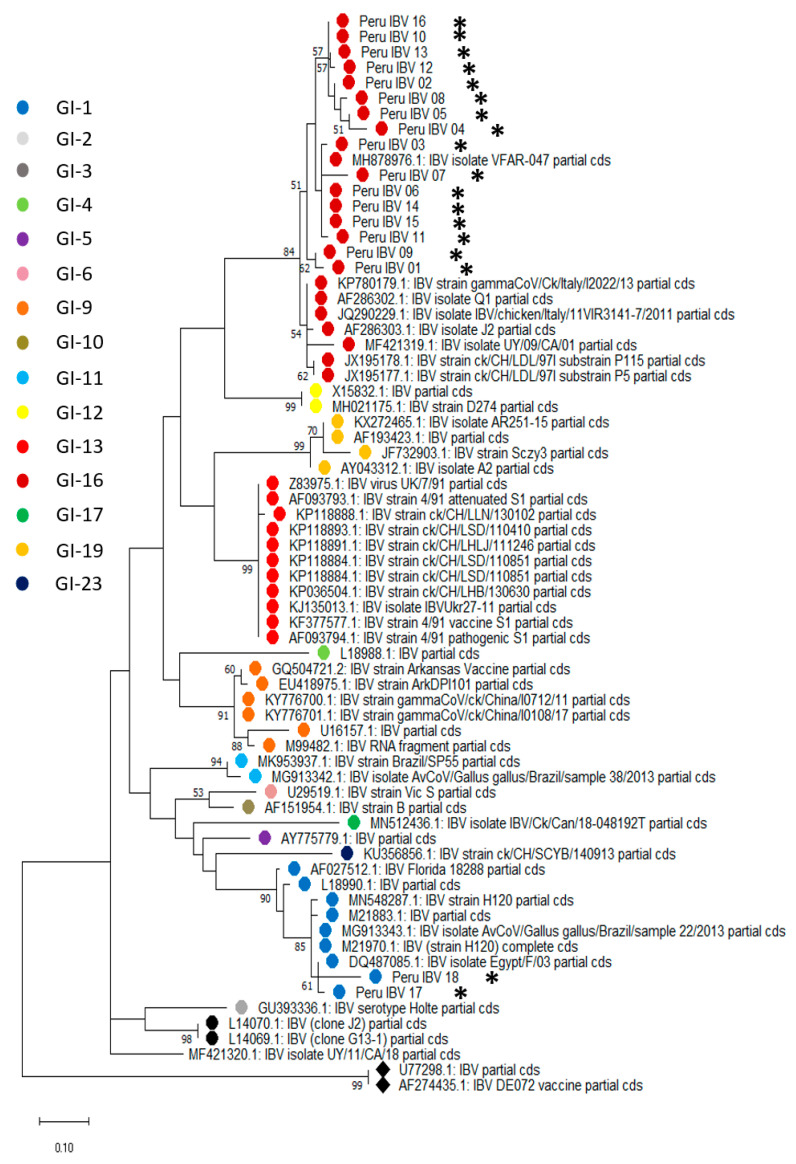
Phylogenetic analysis reveals GI-16 and GI-1 circulation in poultry farms in Peru. Isolated samples were processed for RNA extraction and a PCR that targeted the hypervariable region III (HVR-III) allowed the identification of GI-16 and GI-1 lineages in Peru. The evolutionary history was inferred by using the maximum likelihood method and a poison model. The percentage of trees in which the associated taxa clustered together is shown next to the branches. A discrete gamma distribution was used to model evolutionary rate differences among sites. The tree is drawn to scale, with branch lengths measured in the number of substitutions per site. This analysis involved 70 amino acid sequences. Asterisks represent the sequences obtained in the current study.

S1 genetic sequence analysis has been proposed as a tool for IBV classification [24]. We used this approach to evaluate the phylogenetic relationship among the IBV isolates circulating in farms tested positive for IBV. For this purpose, we collected representative S1 sequences of IBV lineages to establish their genetic relationship. Phylogenetic analyses showed that 16 isolates out of 18 samples sequenced clustered with the infectious bronchitis virus sequences corresponding to lineage 16 within genotype I (GI-16). In contrast, the remaining two samples grouped with lineage 1 in genotype I (GI-1) belonged to the vaccine genotype used in Peru. These two samples were obtained from farms located in the Amazon region (San Martin) in Peru, while the formers were obtained from coastal areas. Genetic distances range from 92.4 to 99.6% of nucleotide identity for the Peruvian isolates within the lineage 16. Conversely, samples 17 and 18, which were grouped within lineage 1, had 99.3 and 96.2% of nucleotide identity with their closest relative, respectively (Supplementary S2, Table S1). Phylogenetic trees at the nucleotide and amino acid levels are shown in Figure 2 and Figure 3. Sequences used for phylogenetic reconstruction are presented in the Appendix A.

### 3.4. Unique Amino Acid Changes Were Detected in the Partial S1 Sequences of the IBV Isolates

**Figure 4:**
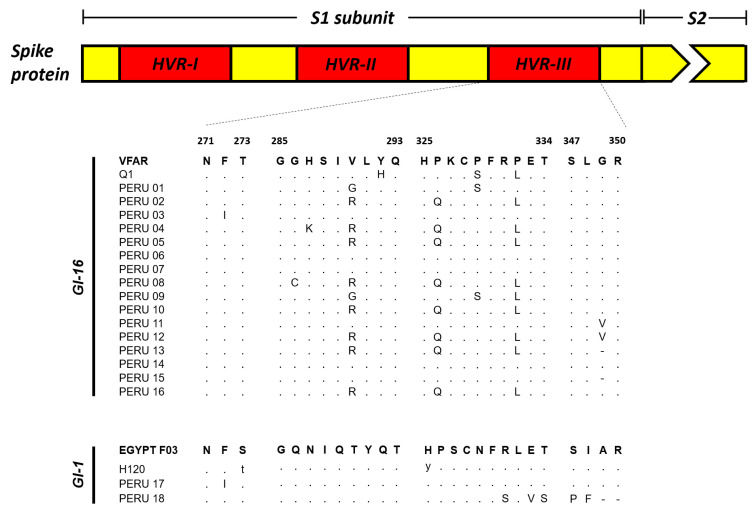
Schematic representation of amino acid changes detected in the IBV belonging to GI-16 and GI-1 lineages: S1 subunit hypervariable region III (HVR-III) of the spike amino acid sequence was compared among the Peruvian isolates and the closest relatives and a representative strain of each GI-16 and GI-1 lineages. Amino acid changes are represented with different letter and those conserved are presented in dots.

As we performed the genetic analysis on the partial S1 region, we were interested in whether nucleotide changes detected represent amino acid changes in the analyzed region. For the sequences clustered in the GI-16 lineage, 10/16 sequences showed changes at the position 290 and 326 compared to their closest relative and a representative of Q1 group. Thus, eight sequences have the amino acid change V290R, while the two remaining are V290G. Furthermore, we found the change P332L in 10/16 sequences analyzed respect to the closest relative, but that was also observed in the GI-1 group. In the GI-1, we identified that sample 18 had the largest amino acid changes within the 347 to 350 aa region. Two amino acid changes (S347P and I348F) and two deletions A349- and R350- were observed. Interestingly, the deletion at position 349 is also present in two samples from the GI-16 lineage, while the other two sequences from the same group have an amino acid change G349V. A schematic representation of these changes is presented in Figure 4.

## 4. Discussion

To date, IB is a major concern in poultry worldwide despite the massive use of vaccination. Thus, our first objective was to identify and characterize the IBV presence in cases of respiratory disease in productive farms. In this context, we analyzed cases of respiratory disease in poultry farms from Lima, Trujillo, Arequipa, and Ica y San Martin during 2015 by a specific and highly sensitive molecular assay-based method. Animals with respiratory signs were submitted for necropsy and tissue collection for diagnostics. In our study, we detected at least one tissue sample per animal positive for IBV by PCR. This evidenced that IBV is, among others, one of the most frequent viral pathogens found on respiratory disease samples in poultry farms in Peru. This was also supported by the viral isolation of selected samples (Ct < 20) in chicken embryonated eggs. This shows that not only the virus was present in those samples, but also that viable virus was recovered from these animals. Hence, our approach indicates that Ct values are useful as approximation methods to determine IBV load for further isolation purposes.

Following viral detection and isolation, we were interested in identifying the circulating IBV lineages. For this purpose, we analyzed the nucleotide and amino acid sequences of a partial S1 region as proposed recently [24]. Spike (S) protein is the most immunogenic and highly polymorphic protein of known coronavirus including IBV. S protein has two structural domains known as S1 and S2 [29,30]. From these, S1 is considered ths most variable region due to the presence of highly variable regions (hypervariable regions, HVRs) that are of relevance for binding function and immune protection [31,32,33,34]. Genetic distances and phylogenetic analysis allowed the identification of the GI-16 lineage circulating in Peru, along with the detection of GI-1 vaccine derived isolates. Interestingly, most isolates belonging to the GI-16 lineage were obtained from poultry farms located in the coastal region of Peru, while those belonging to GI-1 lineage (two out of three) were sampled from the tropical area (San Martin). Although these results do not reflect the frequency of lineage circulation, they might suggest a distinctive IBV circulation pattern in Peru. In this regard, we speculate that the environment might be contributing factor to a differential pattern in IBV lineage circulation. San Martin, a department located in the Amazon region, is separated from the coastal side for the Andean mountains, which create two distinct environments in terms of climate factors and elements, such as humidity, temperature, precipitations, etc. Thus, these factors might have implications in viral transmission across the country. In this regard, a different experimental design will allow us to estimate the prevalence of GI-16 or even other lineages. Despite these genetic differences, we must mention that non-significant differences in IBV clinical presentation were observed in the animals analyzed.

Although we detected differences between GI-16 and GI-1 lineages in the current study, these viruses appear highly conserved, with genetic identity above 96% and 92%, respectively, within each group. These findings suggest that, despite frequent circulation, there are no recombination events occurring within these viruses. Coronavirus recombination is a commonly known mechanism for viral evolution [7,34]. Thus, genetic surveillance is pivotal to address major IBV changes that could affect vaccine effectiveness. In our study, we used a 398-nucleotide segment for phylogenetic reconstruction and might not reveal a complete exploration of other mechanisms underlying viral evolution, such as recombination events. Nevertheless, we are confident that the sequence size analyzed offers a clear perspective into the phylogenetic relationship among other IBV isolates. Furthermore, we identified amino acid changes within the analyzed segment. Thus, our results suggest these changes might be a contributing factor for vaccine evasion, considering the high rates of respiratory diseases due to IBVs. Further studies are required to confirm whether these changes are responsible for the lack of vaccine protection.

Since Massachusetts serotype is the base of vaccines used in most South American countries, and the only vaccine approved for use in Peru, our results indicate that GI-1 lineage isolates obtained in the present study have vaccine origin. This is also possible since the embryonic lesions for these isolates were observed at the second passage, which is common for vaccine-adapted IBV isolates. On the other hand, it is known that viral lineages can be introduced using live IBV vaccines, as might have occurred in other regional countries.

The protectotype hypothesis implies the protection provided by a vaccine against heterologous IBV strains based on conserved antigenic regions in the vaccine and challenge viruses [35,36]. Although these vaccines have been widely used, it remains unclear whether its use is enough to control infections of other distant lineages. Thus, our findings evidence that vaccination protocols need to be adapted to the circulating viruses. Hence, this study might be the first step to make informed decisions from the national health authorities and review the vaccine use to adapt them to circulating lineages. Further studies are needed to determine whether the use of GI-16 vaccines will confer a sustained protection against IBV on farms. Since this study reflects the viral circulation during 2015, it is highly likely that other lineages might be circulating currently. Thus, this study is relevant to start a periodic and sustained genetic surveillance on IBV in areas where its highly prevalent.

## 5. Conclusions

The genetic characterization of the S1 hypervariable region of IBV in Peru determined the GI-16 lineage circulation and that the virus has gained some changes in a relevant portion of the spike protein. Thus, our data show that vaccination programs should be constantly updated to the current circulating virus in areas where the virus is highly prevalent. In this respect, additional studies are required to determine whether other lineages are present and if those are relevant for the efficiency of vaccination programs in Peru.

## Figures and Tables

**Table 1:** General information of primers used for 3′ UTR detection and S1 gene sequencing.

Name	Nucleotide Sequence	Gene	Authors
UTR 41+	5′-ATGTCTATCGCCAGGGAAATGTC-3′	3 UTR	Cavanagh et al., 2002
UTR 31−	5′–GGGCGTCCAAGTGCTGTACCC-3′	3 UTR	Cavanagh et al., 2002
XCE1	5′-CACTGGTAATTTTTCAGATGG-3′	S1	Cavanagh et al., 1999
XCE2	5′-CTCTATAAACACCCTTACA–3′	S1	Cavanagh et al., 1999

**Table 2:** Sample information for S1 sequencing.

ID	Sample Type	Geographical Regions
1	Trachea, lungs	Trujillo
2	Trachea, lungs	
3	Cecal tonsils	
4	Trachea, lungs	Arequipa
5	Trachea, lungs	
6	Cecal tonsils	
7	Trachea, lungs	Lima
8	Kidneys	
9	Cecal tonsils	
10	Kidneys	
11	Cecal tonsils	
12	Cecal tonsils	Ica
13	Cecal tonsils	
14	Trachea, lungs	
15	Kidneys	
16	Cecal tonsils	San Martin
17	Trachea, lungs	
18	Kidneys	

## Data Availability

All data generated or analysed during this study are included in this published article and its supplementary information files. Accession numbers are listed: OM643589, OM674669, OM674670, OM674671, OM674672, OM674673, OM674674, OM674675, OM674676, OM674677, OM674678, OM674679, OM674680, OM674681, OM674682, OM674683, OM674684, and OM674685.

## Supplementary Materials

The following supporting information can be downloaded at: https://www.mdpi.com/article/10.3390/microorganisms11030691/s1, Supplementary File S1, Supplementary File S2: Table S1. Estimates of Evolutionary Divergence between Sequences, References [37,38] are cited in supplementary materials.

## Appendix A

**Table A1:** Information of nucleotide sequences used for phylogenetic analysis.

	Full Name	Genotype-Lineage	Accession Number
1	Avian infectious bronchitis virus DE072 vaccine spike glycoprotein S1 subunit (spike) gene partial cds	GII	AF274435.1
2	Avian infectious bronchitis virus spike glycoprotein S-1 subunit gene partial cds	GII	U77298.1
3	Infectious bronchitis virus isolate UY/11/CA/18 complete genome	GI-11	MF421320.1
4	Avian infectious bronchitis virus (clone G13-1) S1 glycoprotein precursor gene partial cds	GI-3	L14069.1
5	Avian infectious bronchitis virus (clone J2) S1 glycoprotein precursor gene partial cds	GI-3	L14070.1
6	Infectious bronchitis virus serotype Holte complete genome	GI-2	GU393336.1
7	Infectious bronchitis virus isolate Egypt/F/03 spike glycoprotein (S1) mRNA partial cds	GI-1	DQ487085.1
8	Avian infectious bronchitis virus (strain H120) peplomeric protein gene encoding the S1 and S2 subunits complete cds	GI-1	M21970.1
9	Avian coronavirus isolate AvCoV/Gallus gallus/Brazil/sample 22/2013 complete genome	GI-1	MG913343.1
10	Avian infectious bronchitis virus S1 and S2 peplomeric protein gene complete cds	GI-1	M21883.1
11	Infectious bronchitis virus strain H120 complete genome	GI-1	MN548287.1
12	Avian infectious bronchitis virus surface glycoprotein (spike S1) mRNA complete cds	GI-1	L18990.1
13	Avian infectious bronchitis virus Florida 18288 spike glycoprotein S1 subunit gene partial cds	GI-1	AF027512.1
14	Infectious bronchitis virus strain ck/CH/SCYB/140913 complete genome	GI-23	KU356856.1
15	Avian infectious bronchitis virus S1 (S1) gene partial cds	GI-5	AY775779.1
16	Infectious bronchitis virus isolate IBV/Ck/Can/18-048192T complete genome	GI-17	MN512436.1
17	Avian infectious bronchitis virus strain B S1 glycoprotein (S1) gene partial cds	GI-10	AF151954.1
18	Avian infectious bronchitis virus strain Vic S S1 glycoprotein gene partial cds	GI-6	U29519.1
19	Avian coronavirus isolate AvCoV/Gallus gallus/Brazil/sample 38/2013 GI-11 complete genome complete genome	GI-11	MG913342.1
20	Infectious bronchitis virus strain Brazil/SP55 complete genome	GI-11	MK953937.1
21	Avian infectious bronchitis virus RNA fragment	GI-9	M99482.1
22	Avian infectious bronchitis virus spike glycoprotein S1 and S2 subunit gene partial cds	GI-9	U16157.1
23	Infectious bronchitis virus strain gammaCoV/ck/China/I0108/17 complete genome	GI-9	KY776701.1
24	Infectious bronchitis virus strain gammaCoV/ck/China/I0712/11 complete genome	GI-9	KY776700.1
25	Infectious bronchitis virus strain ArkDPI101 complete genome	GI-9	EU418975.1
26	Infectious bronchitis virus strain Arkansas Vaccine complete genome	GI-9	GQ504721.2
27	Avian infectious bronchitis virus surface glycoprotein (spike S1) mRNA 5 end of cds	GI-4	L18988.1
28	Avian infectious bronchitis virus strain 4/91 pathogenic S1 spike glycoprotein gene partial cds	GI-13	AF093794.1
29	Infectious bronchitis virus strain 4/91 vaccine complete genome	GI-13	KF377577.1
30	Infectious bronchitis virus isolate IBVUkr27-11 complete genome	GI-13	KJ135013.1
31	Infectious bronchitis virus strain ck/CH/LHB/130630 complete genome	GI-13	KP036504.1
32	Infectious bronchitis virus strain ck/CH/LBJ/140413 complete genome	GI-13	KP118881.1
33	Infectious bronchitis virus strain ck/CH/LSD/110851 complete genome	GI-13	KP118884.1
34	Infectious bronchitis virus strain ck/CH/LHLJ/111246 complete genome	GI-13	KP118891.1
35	Infectious bronchitis virus strain ck/CH/LSD/110410 complete genome	GI-13	KP118893.1
36	Infectious bronchitis virus strain ck/CH/LLN/130102 complete genome	GI-13	KP118888.1
37	Avian infectious bronchitis virus strain 4/91 attenuated S1 spike glycoprotein gene partial cds	GI-13	AF093793.1
38	Avian infectious bronchitis virus UK/7/91 S gene S1 domain	GI-13	Z83975.1
39	Avian infectious bronchitis virus isolate A2 S1 protein (S1) gene partial cds	GI-19	AY043312.1
40	Infectious bronchitis virus strain Sczy3 complete genome	GI-19	JF732903.1
41	Avian infectious bronchitis virus S1 spike protein (S1) gene partial cds	GI-19	AF193423.1
42	Infectious bronchitis virus isolate AR251-15 complete genome	GI-19	KX272465.1
43	Avian coronavirus strain D274 complete genome	GI-12	MH021175.1
44	Infectious Bronchitis Virus genomic RNA for spike precursor protein S1 and S2 (peplomer subunits S1 and S2)	GI-12	X15832.1
45	Infectious bronchitis virus strain ck/CH/LDL/97I substrain P5 complete genome	GI-16	JX195177.1
46	Infectious bronchitis virus strain ck/CH/LDL/97I substrain P115 complete genome	GI-16	JX195178.1
47	Infectious bronchitis virus isolate UY/09/CA/01 complete genome	GI-16	MF421319.1
48	Avian infectious bronchitis virus isolate J2 spike glycoprotein 1 gene partial cds	GI-16	AF286303.1
49	Infectious bronchitis virus isolate IBV/chicken/Italy/11VIR3141-7/2011 spike glycoprotein S1 (S1) gene partial cds	GI-16	JQ290229.1
50	Avian infectious bronchitis virus isolate Q1 spike glycoprotein 1 gene partial cds	GI-16	AF286302.1
51	Infectious bronchitis virus strain gammaCoV/Ck/Italy/I2022/13 complete genome	GI-16	KP780179.1
52	Infectious bronchitis virus isolate VFAR-047 complete genome	GI-16	MH878976
